# Safety profile of intravenous digoxin in Chinese patients with acute heart failure with reduced ejection fraction: a small-scale prospective cohort study

**DOI:** 10.3389/fphar.2023.1291896

**Published:** 2023-11-13

**Authors:** Xintian Liu, Haojie Zhang, Wenlin Cheng, Qingkun Fan, Zhibing Lu, Xuan Zheng, Gangcheng Zhang

**Affiliations:** ^1^ Center of Structural Heart Disease, Zhongnan Hospital of Wuhan University, Wuhan, China; ^2^ Department of Cardiology, Zhongnan Hospital of Wuhan University, Wuhan, China; ^3^ Laboratory Medicine, Wuhan Asia Heart Hospital, Wuhan University of Science and Technology, Wuhan, China

**Keywords:** acute heart failure with reduced ejection fraction, intravenous digoxin, digoxin toxicity, serum digoxin concentration (SDC), safety evaluation

## Abstract

**Background:** Adverse effects of intravenous digoxin vary from patients and disease status, which should be closely monitored.

**Aims:** To explore the safety profile of intravenous digoxin in acute heart failure with reduced ejection fraction (HFrEF) among Chinese patients.

**Methods:** A clinical prospective, single-center, single-arm, open-label exploratory clinical trial was performed in patients with acute HFrEF at Wuhan Asia Heart Hospital. A fixed dose of 0.5 mg digoxin was used intravenously once per day for 3 days. The normalized dosage of digoxin (NDD), toxic serum digoxin concentration (SDC), and adverse reactions of intravenous digoxin were recorded.

**Results:** A total of 40 patients were recruited in the study. The SDC increased from 1.03 ± 0.34 ng/mL to 1.95 ± 0.52 ng/mL during treatment. 50% (20/40) patients reached a toxic SDC of 2.0 ng/mL, and toxic effects were seen in 30% (12/40) patients. Estimated glomerular filtration rate (eGFR) < 60 mL/min [HR: 5.269; 95% CI: 1.905–14.575, *p* = 0.001], NDD ≥7 μg/kg [HR: 3.028; 95% CI: 1.119–8.194, *p* = 0.029], and ischemic cardiomyopathy [HR: 2.658; 95% CI: 1.025–6.894, *p* = 0.044] were independent risk factors for toxic SDC. Toxic SDC was effectively identified [area under the receiver operating characteristic (ROC) curve = 0.85, *p* < 0.001] using this model, and patients would have a higher risk of toxicity with more risk factors.

**Conclusion:** Intravenous digoxin of 0.5 mg was safe and effective for initial dose but not suitable for maintenance treatment in Chinese patients with acute HFrEF. Patients who had lower eGFR, received higher NDD, and had ischemic cardiomyopathy should be closely monitored to avoid digoxin toxicity.

## Introduction

Digoxin has been known as the only inotropic medicine that slows the heart rate while strengthening cardiac contractility for patients with heart failure (Digitalis Investigation Group, 1997). However, the broad spectrum of toxicity effects from mild sight change to fatal arrhythmias raises concerns during clinical application (Adams et al., 2002; Flory et al., 2012; Grześk et al., 2018; Kapelios et al., 2022). The pharmacokinetics of digoxin is affected by multiple factors such as age, gender, renal function, and complications. Therefore, debates on digoxin safety and effects regarding the narrow therapeutic window, serum digoxin concentration (SDC) of 0.8–2.0 ng/mL, are held in different subgroups (Gona et al., 2023).

Many drugs have shown different pharmacokinetics in Chinese patients because of their different clinical characteristics including low body weight and many complications. Intravenous digoxin is more often used in patients with acute heart failure because of its fast working, which also increases the risk of having toxicity effects. Since Chinese patients started intravenous digoxin treatment since late of 2019, neither optimal therapeutic SDC range nor toxic SDC is well studied. Whether the current dose recommendations and safety evaluations can be applied among Chinese patients remains unclear. Furthermore, acute heart failure with reduced ejection fraction (HFrEF) is critical and often accompanied by multiple-organ dysfunction. The issue of how to use digoxin safely and reduce the occurrence of digoxin toxicity among Chinese patients with acute HFrEF is particularly important.

Here, we sought to investigate the optimal intravenous digoxin dose as well as the safety profile in the setting of acute HFrEF among Chinese patients. We also explore the underlying risk factors predicting toxic SDC of digoxin.

## Methods

### Subjects

Patients with acute HFrEF at the Critical Care Center (CCU) of Wuhan Asia Heart Hospital were consecutively enrolled in this study from July to October 2022. To be eligible for inclusion, the patients had to meet the following criteria: (I) aged between 18 and 90 years; (II) with body weight ≥50 kg; (III) presenting with acute HFrEF; (IV) estimated glomerular filtration rate (eGFR) ≥ 30 mL/min; and (V) baseline SDC <0.5 ng/mL. Patients were excluded if they met any of the following criteria: (I) having a contraindication for digoxin, such as abnormal blood potassium level, hypertrophic cardiomyopathy, aortic stenosis, intracardiac thrombosis, pre-excitation syndrome, acute myocardial infarction, bradyarrhythmias, ventricular arrhythmia, or thyroid dysfunction; (II) receiving invasive mechanical ventilation; (III) with cardiogenic shock; and/or (IV) having oliguria, anuria, or receiving renal replacement therapy.

This study was approved by the Ethics Committee of Wuhan Asia Heart Hospital (No. 2023-YXKY-P004) and was conducted in accordance with the principles described in the Declaration of Helsinki. Written informed consent was obtained from all participants.

### Research protocol

This was a clinical prospective, single-center, single-arm, open-label exploratory clinical trial. The half-life of digoxin was 36 h, and the concentration gradually increased during daily application. Therefore, we adopted a continuously daily intravenous fixed-dose (0.5 mg) 3-day treatment approach given on day 0, day 1 and day 2. The intravenous digoxin (Southwest Pharmaceutical Co., Ltd) of 0.5 mg was diluted to 10 mL with normal saline and pumping at a rate of 1 mL/min. Digoxin levels were measured before the dose of intravenous digoxin were given on day 0, day 1 and day 2 as well as measured on day 3 around 24 h after the last dose was given. SDC >2.0 ng/mL was set as a cut-off value of toxic SDC to stop the subsequent administration due to the increasing chance of digoxin toxicity at maximum therapeutic concentration. Other treatments were applied in accordance with illness conditions and guidelines. Toxicity effects were defined as follows: (I) arrhythmia: atrioventricular blockade, bradycardia, ventricular arrhythmias; (II) gastrointestinal symptoms: anorexia, nausea, vomiting, diarrhea, abdominal pain; (III) visual abnormality: blurred vision, yellow vision, green vision; and (IV) nervous system symptoms: headache, dizziness, insomnia, lethargy, delirium. Severe adverse effects were defined as follows: persistent ventricular tachycardia, ventricular fibrillation, severe bradycardia (heart rate <40 beats/min), and the use of temporary pacemakers.

### Data collection

Baseline clinical characteristics of the enrolled patients were collected, including age, sex, weight, atrial fibrillation, HFrEF etiology, New York Heart Association (NYHA) functional class, vital signs on admission, echocardiography, renal function, and N-terminal pro B-type natriuretic peptide (NT-proBNP). Medicines related to heart failure, and non-pharmaceutical therapies, including percutaneous coronary intervention, non-invasive positive pressure ventilation, intra-aortic balloon pump, radiofrequency catheter ablation for atrial fibrillation, implantable cardioverter defibrillator, as well as cardiac resynchronization therapy, were also collected on admission. SDC, electrolytes (potassium, sodium, magnesium, and chlorine), electrocardiogram (heart rate, PR interval, QRS duration, and QTc interval), and adverse effects were monitored daily during the study.

### Statistical analysis

The normally distributed data are expressed as the mean ± standard deviation. The non-normally distributed data are expressed as the median [interquartile range (IQR): Q1, Q3]. The count data are expressed as the number of cases (percentage). Data were compared with Student’s t-test or Wilcoxon signed-rank test for continuous variables depending on the normality of their distributions. Cox regression analysis was performed to investigate the association between relevant risk factors and toxic SDC. The Kaplan-Meier survival curve was used to illustrate the dynamic change of SDC in patients with different numbers of independent risk factors. The statistical analysis was performed using GraphPad Prism 9.0 (GraphPad, La Jolla, CA, United States). A *p*-value <0.05 was considered to be statistically significant.

## Results

### Characteristics of the enrolled patients

The baseline characteristics of the 40 patients with acute HFrEF are shown in Table 1. The mean age of patients was 64.1 ± 13.6 years, 67.5% of whom were male. 62.5% of the patients presented with atrial fibrillation on admission. 75% had severe impairment on heart function at admission, as evident by elevated heart rate, increased left ventricular diameter, reduced ejection fraction, and elevated NT-proBNP. Notably, the eGFR in more than 90% of patients was <90 mL/min (37/40, 92.5%), and nearly half of these patients had an eGFR <60 mL/min.

**TABLE 1:** Baseline characteristics of Chinese patients with acute HFrEF enrolled in this study (n = 40).

Index	Results
**Age (years)**	64.1 ± 13.6
**Sex**, n (%)	
Male	27 (67.5)
Female	13 (32.5)
**Weight (kg)**	68.2 ± 9.6
**Atrial fibrillation, n (%)**	25 (62.5)
**The cause of HFrEF**, n (%)	
Dilated cardiomyopathy	20 (50.0)
Ischemic cardiomyopathy	20 (50.0)
**NYHA functional class**, n (%)	
Level III	10 (25.0)
Level IV	30 (75.0)
**Vital signs on admission**	
Heart rate (counts per minute)	102.2 ± 29.2
Systolic pressure (mmHg)	132.2 ± 26.9
Diastolic blood pressure (mmHg)	81.5 ± 16.6
**Echocardiogram**	
Left ventricular end-diastolic diameter (cm)	6.5 ± 0.7
EF (%)	27.1 ± 6.4
Albumin (g/L)	37.8 ± 3.3
eGFR (mL/min)	63.1 ± 19.4
**eGFR grouping**, n (%)	
30–59 mL/min	18 (45.0)
60–89 mL/min	19 (47.5)
≥90 mL/min	2 (5.0)
≥120 mL/min	1 (2.5)
**NT-proBNP (pg/mL)**	5,445 (2,227–7,561)

The data are expressed as the mean ± standard deviation, quantity (percentage), or median (interquartile range). HFrEF, heart failure with reduced ejection fraction; NYHA, new york heart association; eGFR, estimation of glomerular filtration rate; EF, ejection fraction; NT-proBNP, amino-terminal brain natriuretic peptide precursor.

### SDC trends

**FIGURE 1:**
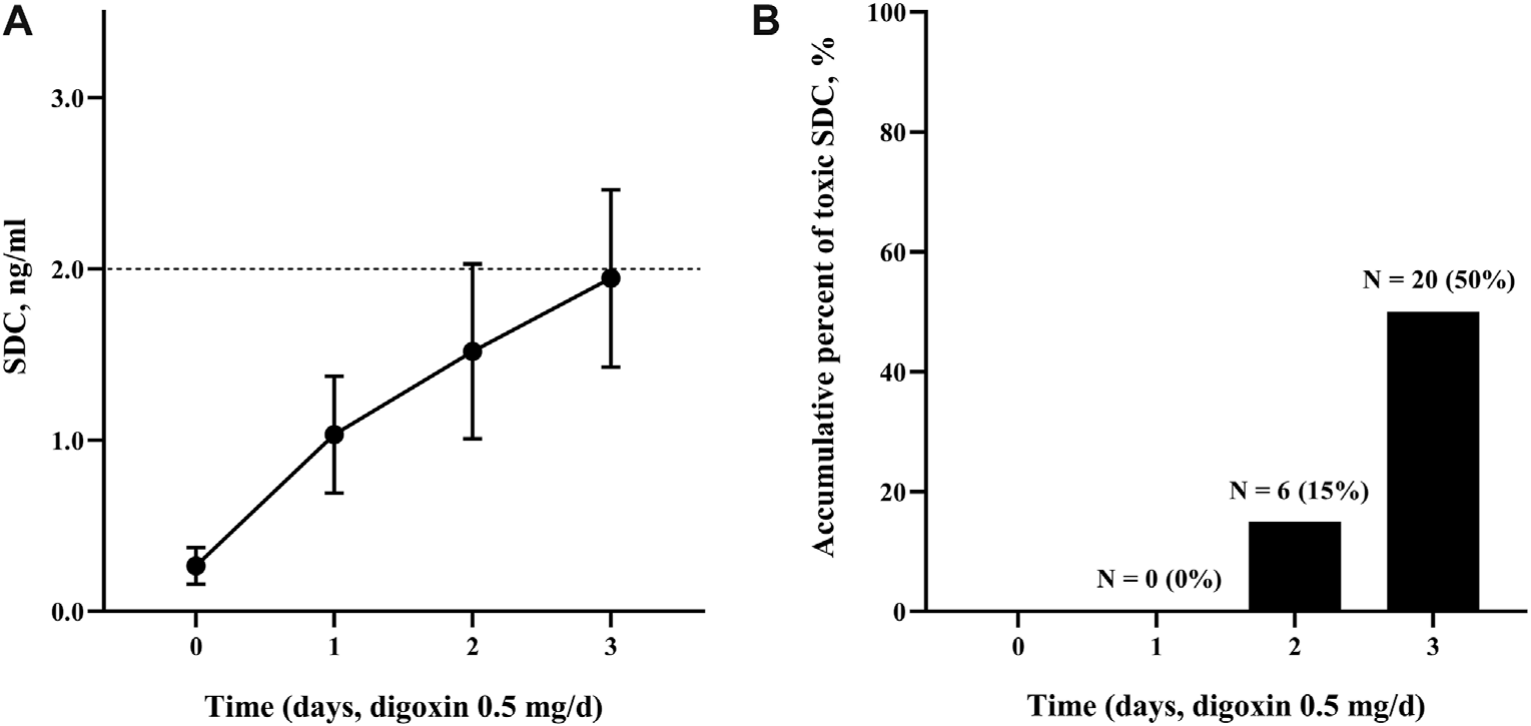
The SDC change and accumulative percent of toxic SDC. **(A)** The SDC in the patients treated with digoxin at an intravenous dose of 0.5 mg daily at the indicated time (0, 1, and 2 days). The cut-off line (dash line) indicated for SDC >2.0 ng/mL. **(B)** The accumulative percentage of toxic SDC. SDC, serum digoxin concentration.

The normalized digoxin dose (NDD) was used to assess the intravenous dosage, which was calculated as the intravenous digoxin application dose divided by body weight. With the application of 0.5 mg intravenous digoxin, mean NDD was 7.2 ± 1.5 μg/kg (range: 4.2–9.8 μg/kg). The baseline SDC was 0.27 ± 0.11 ng/mL. The SDC continuously increased as the daily administration of fixed-dose intravenous digoxin (Figure 1A). Six (15%) patients had a SDC >2.0 ng/mL on the second day, thus the subsequent third dosage was not administered. The remaining patients (34/40, 85%) continued their treatment, and their SDC reached 1.96 ± 0.52 ng/mL on the third day. 41.7% (14/34) of patients who completed 3-day treatment had a SDC >2.0 ng/ml. As shown in Figure 1B, a total of 20 patients had toxic SDC (>2.0 ng/mL). However, all toxic SDC were reduced to below 2.0 ng/mL after drug withdrawal for 24 h.

### Risk factors for toxic SDC

**FIGURE 2:**
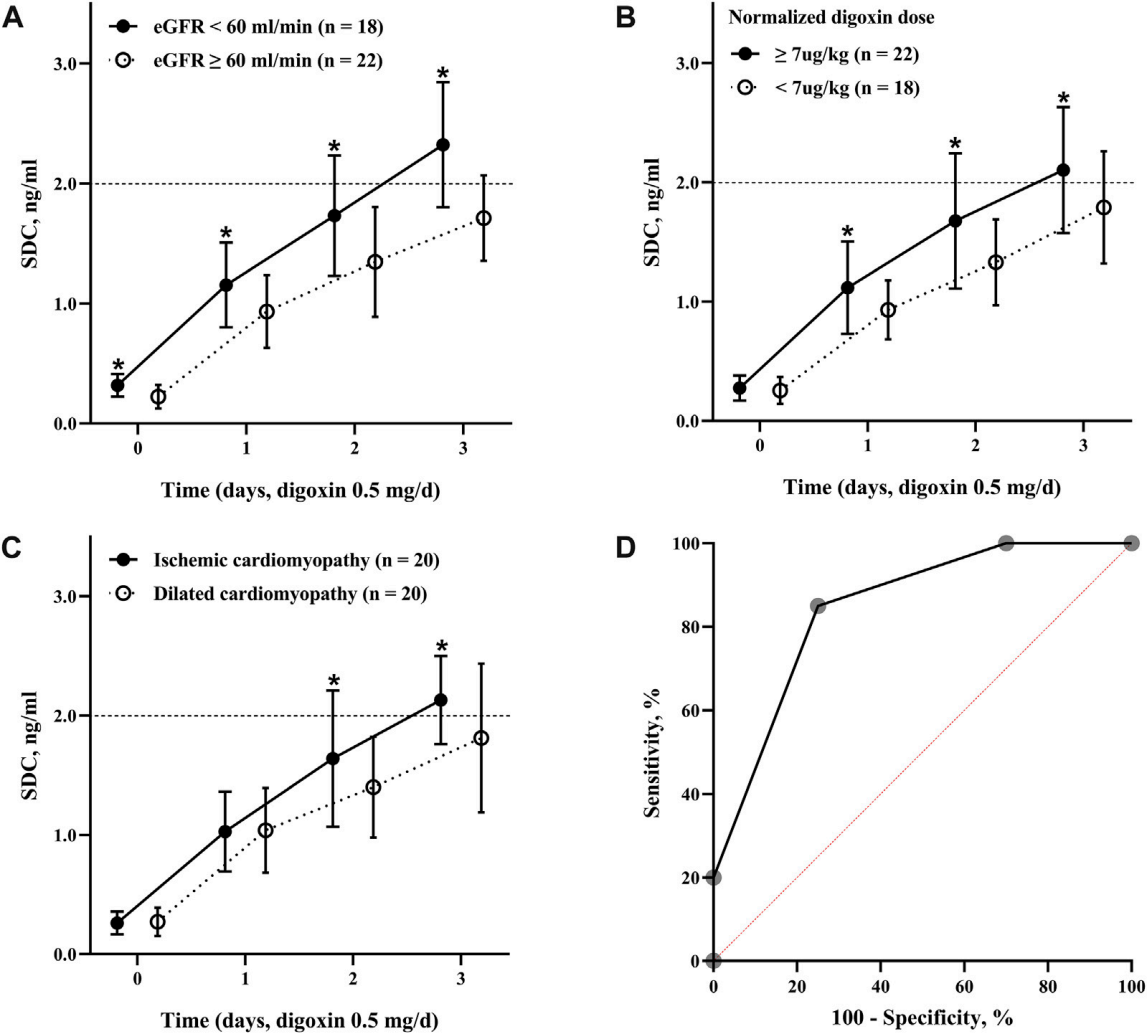
Dynamic changes of SDC with different risk factors. **(A)** The SDC in patients with an eGFR over or below 60 mL/min; **(B)** the SDC in patients received digoxin at a dose over or below 7 μg/kg; **(C)** the SDC in patients diagnosed with dilated cardiomyopathy or ischemic cardiomyopathy; **(D)** the ROC curve of toxic SDC predicted by the number of independent risk factors. The area under the ROC curve was 0.85, *p* < 0.001. *, *p* < 0.05. SDC, serum digoxin concentration; eGFR, estimation of glomerular filtration rate; ROC curve, receiver operating characteristic curve.

**FIGURE 3:**
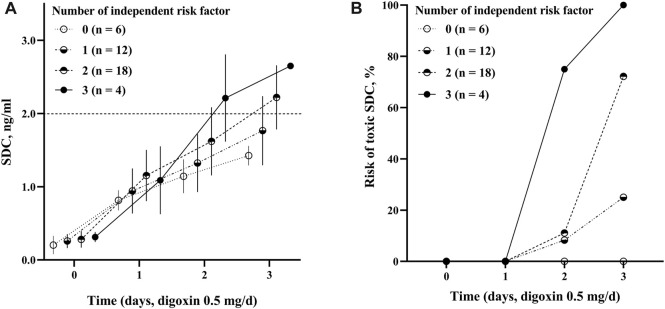
The positive association between the risk factors and SDC. **(A)** The change of SDC with different number of risk factors. The dashed line parallel to the horizontal axis (2.0 ng/mL) indicated for toxic SDC; **(B)** the risk of toxic SDC based on different number of risk factors. Overall difference between groups log-rank = 18.23, *p* < 0.001. SDC, serum digoxin concentration.

Table 2 showed Cox analysis of risk factors for toxic SDC which was defined as SDC >2.0 ng/mL in our study. Among the factors, body weight was a protective factor to reduce the risk of reaching toxic SDC. In the multivariate analysis, ischemic cardiomyopathy, a NDD ≥7 μg/kg, and an eGFR <60 mL/min significantly increased the risk of accumulation of SDC by 2 to 5 folds separately (Figures 2A–C). Using this multi-factor model, toxic SDC would be effectively identified [area under the receiver operating characteristic (ROC) curve = 0.85, *p* < 0.001, Figure 2D]. Furthermore, the K-M curve showed that the risk for toxic SDC increased gradually as the number of independent risk factors increased (Figure 3A). Patients with 3 risk factors had nearly 5 folds higher risk of reaching toxic SDC in the third day of application (Figure 3B, log-rank = 18.23, *p* < 0.001). Interestingly, the first dosage of intravenous digoxin would not introduce toxic SDC regardless of their baselines.

**TABLE 2:** Risk factors of toxic SDC (Cox regression analysis).

Variables	Univariate			Multivariate		
	HR	95% CI	P	HR	95% CI	P
Age (years)	1.046	1.005–1.088	0.026			
Sex						
Female	3.937	1.618–9.582	0.003			
Male	1.000					
Weight (kg)	0.965	0.939–0.998	0.047			
HFrEF etiology						
Ischemic cardiomyopathy	2.018	1.005–5.059	0.034	2.658	1.025–6.894	0.044
Dilated cardiomyopathy	1.000					
eGFR						
<60 mL/min	3.478	1.335–9.065	0.001	5.269	1.905–14.575	0.001
≥60 mL/min	1.000					
NT-proBNP(10 (Flory et al., 2012) pg/mL)	1.084	1.028–1.114	0.003			
Normalized digoxin dose						
≥7 μg/kg	2.201	1.045–5.732	0.016	3.028	1.119–8.194	0.029
<7 μg/kg	1.000					

Normalized digoxin dose was calculated by digoxin application dose divided by body weight. SDC, serum digoxin concentration; HFrEF, heart failure with reduced ejection fraction; eGFR, estimation of glomerular filtration rate; NT-proBNP, amino-terminal brain natriuretic peptide precursor; HR, hazards ratio; CI, confidence interval.

### Toxicity effects and outcomes

A total of 22 adverse reactions in 12 patients were observed during the study, including 5 nausea and vomiting, 2 abdominal pain, 2 delirium, 4 frequent premature ventricular beats, 4 short ventricular tachycardia, 4 bradycardia, 1 rash. No severe adverse effects such as persistent ventricular tachycardia, ventricular fibrillation, severe bradycardia, and the use of temporary pacemakers were recorded.

Patients showed significantly improvement at the end of the study. The heart rates reduced from 98 ± 23 to 78 ± 18 beats/min (*p* < 0.001). The trends of electrocardiogram parameters (heart rates, PR interval, QRS duration, and QTc interval) and parameters in the electrolytes were listed in Supplementary Figures S1, S2, respectively. NT-proBNP was significantly lower than those at the baseline [5,445 (2,277–7,561) vs 2,193 (854–5,108) pg/mL, *p* < 0.001]. The median length of hospitalization for the enrolled patients was 9 [6–12] days. The major treatments except digoxin based on clinical condition were listed in Supplementary Table S1.

## Discussion

The current study demonstrated the following important findings: (I) after the administration of the fixed dosage of intravenous digoxin (0.5 mg/day) for 3 consecutive days, the SDC of the Chinese patients with acute HFrEF gradually increased from 1.02 to 1.96 ng/mL. The percentage of patients in whom the SDC >2.0 ng/mL increased with time; (II) the eGFR, NDD, and ischemic cardiomyopathy were the 3 important independent risk factors that associated with toxic SDC. The hazard ratios of the toxic SDC in the patients with an eGFR <60 mL/min, NDD ≥7 μg/kg, and ischemic cardiomyopathy were 5.269, 3.028, and 2.658, respectively; and (III) the risk of toxic SDC was higher in those whom with more risk factors as SDC increased rapidly.

In previous studies and guidelines, the loading digoxin dose of 10 μg/kg is recommended and can be added up to 1.0 mg or even 1.5 mg in total (Adams et al., 2002; McDonagh et al., 2021). However, 15% or 50% patients reached toxic SDC with the accumulation of 1.0 or 1.5 mg in total after treating for 2 or 3 days in our study. We also found the SDC was maintained under 2.0 ng/mL when using 0.5 mg of intravenous digoxin as loading dose. In fact, the recommended digoxin level for management of chronic heart failure was 0.5–1.0 ng/mL (Hunt et al., 2009), and in the present study about 1.0 ng/mL can be achieved by one single loading dose of intravenous 0.5 mg digoxin. Therefore, a lower loading dose of intravenous digoxin, 0.5 mg in our study, would be suggested for Chinese patients with acute HFrEF to avoid toxicity while achieving the possible therapeutic level for heart failure management.

A maintenance dosage of 0.125–0.5 mg regardless of individual differences is suggested to achieve the treatment effects while avoiding digoxin toxicity. Our study showed that 50% of patients had relatively safe SDC while the others had a toxic SDC during digoxin administration. To investigate the underlying factors causing this phenomenon, a Cox regression analysis was performed, and the results showed that an eGFR <60 mL/min, NDD ≥7 μg/kg, and ischemic cardiomyopathy were independent risk factors associated with toxic SDC.

It is well documented that renal insufficiency affects digoxin concentration (Flory et al., 2012; Pawlosky et al., 2013; Sc et al., 2017; Bavendiek et al., 2023). In the current study, more than 90% of the patients with acute HFrEF had an eGFR <90 mL/min, and nearly half of the patients had an eGFR <60 mL/min. Thus, attention needs to be paid to renal function to prevent the administration of excessive doses of digoxin. Myocardial ischemia is another reason affecting SDC. Myocardial ischemia inhibits activation of Na^+^-K^+^ ATPase in the cell membrane, which promotes the accumulation of digoxin in myocardial cells and causes toxicity (Rogers et al., 2010; Ambrosy et al., 2018; McDonagh et al., 2021). Our study showed that the chance of SDC accumulation and the risk of reaching toxic SDC increased significantly as the number of independent risk factors increased. The SDC was maintained at a safe level for those who had no risk factors, even when the accumulative intravenous digoxin reached 1.5 mg after 3-day application. Conversely, all patients with 3 independent risk factors reached toxic SDC after 2 or 3-day application. These results suggest that the more independent risk factors a patient has, the less of digoxin dose should be applied on the basis of the loading dose. Otherwise, the intravenous digoxin could be administered every other day and adjusted according to the SDC to avoid the risk of digoxin toxicity.

Notably, we used a fixed-dose application in the present study to explore the safety profile of intravenous digoxin. As the results from our study, an intravenous digoxin of 0.5 mg would be suggested as the loading dose for similar patients, and the maintenance doses should be adjusted regarding their risk factors and the SDC monitoring results. In this way, the SDC range can be effectively controlled for even a longer-term therapy, and the risk of digoxin toxicity can be greatly reduced while maintaining the therapeutic concentration of digoxin.

The application of intravenous digoxin was relatively safe in this study. The maximum SDC was only 3.06 ng/mL, and the SDC in all the patients in whom having toxic SDC decreased to <2.0 ng/mL after 24 h of treatment termination. Only a few patients suffered from mild toxicity effects, and no persistent ventricular tachycardia and severe bradycardia were observed like previous studies reported (Hornestam et al., 1999; Hood et al., 2004). These findings suggest that it is very important to monitor the SDC closely during the administration of intravenous digoxin and observe the clinical manifestations of patients during treatment. It has been recommended to maintain a SDC no more than 1.2 ng/mL during digoxin application to minimize the incidence of toxicity effects (Gona et al., 2023). Although we observed the highest SDC in our study was 2.01 ± 0.52 ng/mL, it is unnecessary to maintain such a high concentration of digoxin in daily clinical practice.

There are some limitations in our study. First, because of the small sample size, these factors appear to be a little too large to be included in the regression analysis (Vittinghoff and McCulloch, 2007), so the statistical results may not be robust and will need to be supported by a larger sample in the future. Second, the target population of this study were patients with acute heart failure with dilated hearts and reduced ejection fraction. It is unclear whether the results would also be applicable to patients with other types of heart failure. Other types of heart failure should be included in further study. Third, the following patients were excluded from the study: elderly patients aged ≥90 years old, patients with a low body weight of <50 kg, patients with severe renal failure (i.e., an eGFR <30 mL/min, oliguria, anuria, or renal replacement therapy), and critically ill patients (i.e., those in cardiogenic shock or those that required invasive mechanical ventilation). Thus, the administration of digoxin in these patients requires further research and digoxin should be administered with caution.

## Conclusion

The study shows that intravenous digoxin is safe for clinical administration under close monitoring. The loading dose of intravenous digoxin for Chinese patients with acute HFrHF is suggested at 0.5 mg, NDD of 4.2–9.8 μg/kg. The application of digoxin should be with caution in vulnerable patients, who are female, the elderly, frail, malnourish, hypo- or hyper-kalaemic, renal dysfunction, and deteriorated cardiac function. Further consideration should be paid on the NDD, eGFR, and heart failure etiology to avoid toxic SDC.

## Data Availability

The raw data supporting the conclusion of this article will be made available by the authors, without undue reservation.
