# Supplementary material for: Safety profile of intravenous digoxin in Chinese patients with acute heart failure with reduced ejection fraction: a small-scale prospective cohort study
Source: Front Pharmacol. 2023 Nov 13;14:1291896. doi: 10.3389/fphar.2023.1291896 (PMC10679729; doi:10.3389/fphar.2023.1291896)
Supplement: Supplementary file 1 [file Table1.DOCX]

**Supplementary**

**Table S1** Major treatments except digoxin based on clinical condition

| **Major treatments** | **N (%)** |
| --- | --- |
| **Medical treatment** |  |
| Diuretics |  |
| Furosemide | 39 (97.5) |
| Spironolactone | 35 (87.5) |
| Tolvaptan | 6 (15.0) |
| Renin-angiotensin-aldosterone system inhibitors |  |
| ACEI | 2 (5.0) |
| ARB | 1 (2.5) |
| ARNI | 33 (82.5) |
| β-receptor blocker | 38 (95.0) |
| Ivabradine | 2 (5.0) |
| Amiodarone | 2 (5.0) |
| Calcium antagonists | 2 (5.0) |
| **Non-medical treatment** |  |
| PCI | 11 (27.5) |
| Non-invasive positive pressure ventilation | 8 (20.0) |
| IABP | 4 (10.0) |
| RFCA for atrial fibrillation | 1 (2.5) |
| ICD | 1 (2.5) |
| CRT/D | 1 (2.5) |

ACEI, angiotensin converting enzyme inhibitor; ARB, angiotensin receptor blocker; ARNI, angiotensin receptor-neprilysin inhibitor; PCI, percutaneous coronary intervention; IABP, intra-aortic balloon pump; RFCA, radio frequency catheter ablation; ICD, cardiac resynchronization therapy with a defibrillator; CRT/D, implantable cardioverter defibrillator.

**Figure S1** Dynamic changes in the parameters in ECG. (A) The heart rate trend. (B) The QRS complex trend. (C) The PR interval trend. (D) The QTc interval trend. ECG, electrocardiogram.

**Figure S2 Dynamic changes in the parameters in the electrolytes.** (A) The serum sodium trend. (B) The serum potassium trend. (C) The serum magnesium trend. (D) The serum chlorine trend. The dashed lines parallel to the horizontal axis indicate the upper and lower limits of each parameter in the electrolytes.
